# Energy and Redox Homeostasis in Tumor Cells

**DOI:** 10.1155/2012/593838

**Published:** 2012-05-30

**Authors:** Marcus Fernandes de Oliveira, Nívea Dias Amoêdo, Franklin David Rumjanek

**Affiliations:** Instituto de Bioquímica Médica, Universidade Federal do Rio de Janeiro, Cidade Universitária, 21941-970 Rio de Janeiro, RJ, Brazil

## Abstract

Cancer cells display abnormal morphology, chromosomes, and metabolism. This review will focus on the metabolism of tumor cells integrating the available data by way of a functional approach. The first part contains a comprehensive introduction to bioenergetics, mitochondria, and the mechanisms of production and degradation of reactive oxygen species. This will be followed by a discussion on the oxidative metabolism of tumor cells including the morphology, biogenesis, and networking of mitochondria. Tumor cells overexpress proteins that favor fission, such as GTPase dynamin-related protein 1 (Drp1). The interplay between proapoptotic members of the Bcl-2 family that promotes Drp 1-dependent mitochondrial fragmentation and fusogenic antiapoptotic proteins such as Opa-1 will be presented. It will be argued that contrary to the widespread belief that in cancer cells, aerobic glycolysis completely replaces oxidative metabolism, a misrepresentation of Warburg's original results, mitochondria of tumor cells are fully viable and functional. Cancer cells also carry out oxidative metabolism and generally conform to the orthodox model of ATP production maintaining as well an intact electron transport system. Finally, data will be presented indicating that the key to tumor cell survival in an ROS rich environment depends on the overexpression of antioxidant enzymes and high levels of the nonenzymatic antioxidant scavengers.

## 1. A Brief Prelude

Every biochemical reaction within living cells involves the transduction of some degree of free energy that is ultimately derived from the oxidation of dietary nutrients. Most of this free energy is made biologically available as reversible phosphorylation reactions involving adenosine triphosphate (ATP) that is continuously being produced and utilized by cells to drive thermodynamically nonspontaneous reactions, such as ion transport, muscle contraction, protein synthesis, and DNA replication. Just as an example of the stupendous biological power, the amount of free energy transduced by our body during light walking is about 3,18 × 10^−3^ W/g, which is roughly 16.000 times more than the fusion reactions that take place in the Sun core [1]. It is known that phosphate esterification into ATP can occur by several processes but the best known are the phosphocreatine-ATP shuttle, glycolysis, and oxidative phosphorylation [2]. Oxidative phosphorylation is capable of producing significantly more ATP per mole of substrate than glycolysis in reactions completely dependent on the availability of oxygen. However, the utilization of oxygen by cells, albeit the advantages of oxidative phosphorylation, is not without consequence, since partially reduced oxygen intermediates, the so-called reactive oxygen species (ROS) play key roles in cellular redox homeostasis [3] that may have a role in tumorigenesis.

## 2. Mitochondria and Bioenergetics

### 2.1. Fermentation, Pasteur, Warburg, and Crabtree

Fermentation was the first metabolic pathway to be fully known, thanks to the key findings of many researchers such as Louis Pasteur, who defined the biological nature of the process and Eduard Buchner, who showed that cell-free extracts could carry out fermentation. Later, Otto Meyerhoff experimentally demonstrated that a process similar to fermentation occurred in skeletal muscles, although generating a different final product, lactate [4]. He also showed that, in the absence of oxygen, glycogen was converted to lactate and when oxygen was present lactate was converted back to glycogen, establishing the cyclic nature of lactate metabolism in muscles (the *lactate shuttle*). In the context of cancer, when cells that use glucose as the main substrate to drive ATP synthesis are subjected to hypoxia, as happens to the cells located in the center of the tumor mass, glucose uptake and metabolism increase significantly in order to maintain cellular ATP levels. Since under limited oxygen availability the oxidative phosphorylation machinery is not fully operational, other pathways are recruited in order to supply the energy demand. The reversible nature of increased glucose uptake and metabolism when cells experiment hypoxia is known as the *Pasteur Effect*. Also relevant is the reversible repressive effect of glucose over respiration, known as the *Crabtree effect* [5]. Thus, in spite of a functional oxidative phosphorylation machinery, most solid tumors exhibit a reversibly switch of their metabolism towards lactic fermentation, even under normoxia. Thus, contrasting with the *Pasteur Effect*, the limitation of respiration in the *Crabtree effect* is not due to oxygen availability, but rather to an acute repressive signaling cascade triggered by glucose over the mitochondrial function. For this reason, sometimes the *Crabtree effect* is also referred to as *Reverse *or* Inverted Pasteur Effect*. However, the molecular mechanisms that underlie the *Crabtree effect* remain elusive. Finally, the long-term metabolic reprogramming that takes place in many cancer cells and which bears on cancer is known as the *Warburg effect* [6]. Otto Warburg observed that cancer cells displayed decreased respiration and enhanced lactate production, suggesting that they depended mainly on fermentative metabolism for ATP generation [7]. It is commonly assumed that tumors manifesting the *Warburg effect* do so because the oxidative phosphorylation machinery is somehow impaired. In this context a growing body of evidence shows in fact that the oxidative phosphorylation is preserved in many cancer cells, as will be discussed in the following section. The point that should be stressed here regarding the main difference between *Crabtree* and *Warburg effects* is that in the former the oxidative phosphorylation is rapidly and reversibly downregulated by the repressive effect of glucose, whereas in the latter, there is a long-lasting irreversible effect favoring fermentation due to the increased expression of proteins involved in glucose transport and metabolism [5].

### 2.2. Mitochondria and the Processes of Energy Transduction

Structurally, mitochondria are organelles enclosed by two very distinct membranes: an outer membrane, moderately selective, and an inner membrane which is protein rich and highly selective. These compartments are structurally and functionally different. The tricarboxylic acid (TCA) cycle enzymes are located within this compartment, whereas the proteins that comprise the electron transport system (ETS) occur in the inner mitochondrial membrane. The redox reactions mediated by different compounds from ubiquinone to iron/copper-sulphur clusters, cytochromes, and finally oxygen reduction to water (respiration) take place at the inner mitochondrial membrane. Recently, an effort to identify the whole set of mitochondrial proteins in different tissues of mice, rat, and human demonstrated that this organelle is composed of almost 1100 different proteins [8].

The ETS is essentially composed of proteins that contain an array of redox centers making up the complexes commonly listed from I to IV [2]. It is important to mention, however, that respiration can be promoted by multiple sites of electrons entry to the ETS in which electrons converge at the ubiquinone reduction (Q-junction) [9]. Importantly, the free energy released during the electrons transport by the ETS complexes is linked to the transport of protons across the inner mitochondrial membrane. Due to its proton impermeable nature, an electrochemical gradient is established [10]. This electrochemical proton gradient has two components; one chemical (ΔpH) and the other electrical (Δ*ψ*) in nature, which together represent the protonmotive force (*pmf*). The free energy accumulated in the form of *pmf* can be converted to chemical energy by means of the complex molecular motor activity of the F_1_F_o_ ATP synthase, which allows the return of protons back to the mitochondrial matrix coupled to ATP production [11]. *pmf* is important not only for ATP synthesis but for many processes such as the control of substrate transport to mitochondrial matrix, respiratory rates [12], calcium homeostasis [13], ROS generation [14] and heat production [15].

### 2.3. Redox Reactions in the ETS

The first ETS redox centers were described in the nineteenth century by Charles MacMunn. In 1883, he found a peculiar pigment (*myohematin*) in the muscle of insects, whose light absorption pattern was quite similar to heme. MacMunn proposed the respiratory nature of these pigments and suggested that they were not derived from hemoglobin since they were found in organisms that knowingly did not have it [16]. Decades later the parasitologist David Keilin revisited the problem using an ingenious device, the microspectrophotometer. During his studies, Keilin found the very same four absorption bands identified by MacMunn not only in the fly, but in *Bacillus subtilis* and in baker's yeast. Keilin called the ubiquitous colored pigments *cytochromes*. Eventually, Keilin also determined that light absorption pattern of the four bands changed distinctly when metabolic poisons were administered, or when yeasts were deprived of oxygen. He concluded that the intensity of light absorption bands resulted from the cytochromes reduction. As a result, it became paramount to understand how cytochromes supported respiration.

 Besides heme-containing cytochromes, it is known today that many distinct redox centers are involved in the electron transport along the ETS such as the iron/copper-sulphur clusters, the flavin-containing enzymes, and ubiquinone. These compounds differ not only in composition, but also in the number of electrons transported and their redox potentials. An interesting feature of the ETS is the presence of two mobile electron transfers: the nonproteic organic molecule ubiquinone (UQ) and cytochrome *c*. Although UQ is quite hydrophobic, it is highly mobile and promotes the bridging between complexes I, II, Glycerol-3 phosphate dehydrogenase, and electron transfer flavoprotein-ubiquinone oxidoreductase with complex III [2]. Unlike the cytochromes and iron/copper-sulphur clusters, UQ can be reduced by two electrons, generating the fully reduced form ubiquinol (UQH_2_). However, during its redox cycle, UQ can be partially reduced, generating an unstable ubisemiquinone (UQ^•−^) radical. Cytochrome *c* is a small heme protein which is loosely bound to the inner mitochondrial membrane and is responsible for the transport of a single electron. Cytochrome *c* also participates as a major inducer of apoptosis, when released by the mitochondria in response to proapoptotic stimuli, such as calcium and oxidative stress conditions [17]. Cytochrome *c* is bound to inner mitochondrial membrane by means of a direct interaction with cardiolipin which can be disrupted when cardiolipin is oxidatively modified in redox imbalance [18].

The ETS complex I, or NADH:ubiquinone oxidoreductase, is considered one of the largest known membrane proteins and can be visualized by electron microscopy, which reveals its characteristic “*L*” shape [19]. The structure of this huge protein complex was recently elucidated [20]. Complex I activity couples the transfer of two electrons from NADH to ubiquinone, in parallel with the translocation of four protons across the inner mitochondrial membrane. This activity provides about 40% of the proton-motive force generation coupled to mitochondrial ATP synthesis. In mammals, complex I contains 45 subunits resulting in an apparent molecular mass of about 1 MDa and it has been implicated in many human neurodegenerative diseases.

Complex II, also known as succinate dehydrogenase, converts succinate to fumarate, which is the only TCA cycle reaction taking place at the inner mitochondrial membrane. The electrons from succinate oxidation directly contribute to UQ reduction and oxidative phosphorylation as well. The elucidation of complex II structure revealed that the architecture of its redox centers is arranged in a way that prevents ROS production at the FAD site [21, 22]. Complex II contains four subunits, two of which are integral membrane, while the other two face the mitochondrial matrix, which contains covalently bound FAD and three iron-sulphur clusters.

Glycerol 3-phosphate dehydrogenase (G3PDH) has two isoforms, the cytosolic (*c*G3PDH) and the mitochondrial (*m*G3PDH). The *m*G3PDH is bound to the inner membrane facing the mitochondrial intermembrane space [23] and transfer electrons generated from dehydrogenation of G3P to UQ. The activity of this enzyme is closely associated to the oxidation of cytosolic NADH from the glycolytic pathway, regenerating the “pool” of NAD^+^ from glycolysis.

The other component of ETS contributing to electrons entry is the electron transfer flavoprotein-ubiquinone oxidoreductase (ETF-QO), which is an intrinsic membrane protein located in the inner mitochondrial membrane. ETF-QO contains an FAD molecule and a [4Fe4S] cluster. The protein represents the only input site of the ETS for electrons derived from nine flavoprotein acyl-CoA dehydrogenases and two N-methyl dehydrogenases, which are eventually transferred to UQ [24]. Incidentally, of the four distinct ETS electrons entry sites, only complex I contributes to the Δp, as it couples the energy of electrons transport to the proton translocation across inner mitochondrial membrane.

Complex III, also called ubiquinol:cytochrome *c* oxidoreductase, couples the transfer of two electrons from UQH_2 _to cytochrome *c* generating a proton gradient across the inner mitochondrial membrane. The redox centers involved in complex III activity are the cytochrome *b*, which has two heme type b (BL and BH), cytochrome *c*_1_, and the *Rieske* iron sulphur protein [2Fe-2S] [25]. Complex III is a dimer. Each monomer consists of 11 different polypeptide subunits yielding a total of 240 kDa [25]. The presence of two active sites in complex III is an essential feature for the operation of Q cycle [26]. One of the sites is responsible for UQH_2_ oxidation and proton translocation to the intermembrane space and is located near the cytoplasmic side of the inner membrane (Qp). The other site is responsible for reducing the UQ, capturing electrons from the inner side of the membrane and is located near the matrix side (Qn). Since UQH_2_ donates two electrons and the cytochromes are reduced by only one, electron transfer from the UQH_2_ to complex III is bifurcated. The first step comprises the oxidation of UQH_2_ at the Qp site of complex III. One of its electrons is transferred to the *Rieske* iron sulphur protein while the other is transported to heme *b*_*L*_. There are two fundamental reasons for the passage of the two electrons from UQH_2_ to cytochrome *c*_1_ and cytochrome *b*. The first, and more obvious, is the fact that UQ is reduced by two electrons and two protons, while cytochrome *c* by a single electron. The other is structural. Because the *Rieske* center is mobile in complex III and directs the electrons to reduce cytochrome *c*, eventually it returns back to the Qp site [27]. Thus, at the same time, the *Rieske* center is close to cytochrome *c*, and distant from the site receiving the Qp of another UQH_2_ electron [25]. During the Q cycle, for each pair of electrons, two protons are consumed from the matrix and four protons are released into the intermembrane space, promoting the net transport of two protons.

The electron present at the cytochrome *c* is then transferred to the terminal ETS complex IV, also known as cytochrome *c* oxidase. The mammalian complex IV is composed of 13 subunits and contains several redox centers such as two hemes, one cytochrome *a* and cytochrome *a*_3_, and two copper centers, the Cu_A_ and Cu_B_ centers. In fact, the site of oxygen reduction to water is composed of a binuclear center which contains cytochrome *a*_3_ and Cu_B_. Complex IV catalyzes the transfer of four electrons from four reduced cytochrome *c* to oxygen, completely reducing it to two water molecules [28]. Oxygen reduction involves a complex redox cycle in which Cu_A_ and Cu_B_ centers, as well as the heme *a*, heme *a*_3_ and a tyrosine residue participate. Firstly, the oxygen molecule binds to the enzyme complex at the heme *a*_3_-Cu_B_ binuclear center on its fully reduced state in the following redox configuration: heme *a*_3_^+2^-Cu_*B*_^+1^. In fact, molecular oxygen binds to the binuclear center at the heme *a*_3_^+2^ site and then the bonds between oxygen atoms are disrupted in such a way that one of the oxygen atoms remains bound to heme *a*_3_ site and the other one, to the Cu_B_ center. During this step, two electrons are transferred from the heme *a*_3_^+2^ to the oxygen atom bound, adopting the heme *a*_3_^+4^ oxidation state (ferryl). The other oxygen atom is reduced by means of transfer of two electrons, one originating from Cu_*B*_^+1^ center, which becomes Cu_*B*_^+2^, and the other one from the tyrosine 244 residue, which is cross-linked to histidine 240 where the Cu_B_ is bound. In the next step, the tyrosine is regenerated with electrons from one reduced cytochrome *c* molecule which is transferred via heme *a*. An additional electron is transferred from one reduced cytochrome *c* molecule, through heme *a*, to the heme *a*_3_^+4^, which converts to its ^+3^ redox state. Complete enzyme regeneration is achieved by further delivery of two electrons, from two reduced cytochrome *c* molecules, through heme *a*, to the active site, restoring the heme *a*_3_^+2^ and Cu_*B*_^+1^ redox configuration and allowing each of the two oxygen atoms originally in oxygen to dissociate as water. Interestingly, the very same site where oxygen binds to the cytochrome *c* oxidase, the heme *a*_3_^+2^ at the binuclear center, is also able to bind other ligands such as cyanide (CN-) carbon monoxide (CO) and nitric oxide (NO), all of which are respiration inhibitors [2].

During the sequential redox reactions of electrons transport along the ETS to their final acceptor, molecular oxygen, a significant part of the energy is conserved as protons are transported from the mitochondrial matrix to the intermembrane space, generating the Δp, specifically at the complexes I, III, and IV. As proton transport is thermodynamically unfavorable, coupling the energy released by the electrons transport at complexes I, III, and IV overcomes the energy barrier.

### 2.4. The Chemiosmotic-Dependent ATP Synthesis

The ETS-dependent proton transport across the inner mitochondrial membrane allows the Δp formation and represents the ultimate energy source by which mitochondrial ATP is synthesized [10]. One of the first proposals was based on the idea that electrons passage through the ETS would release a finite amount of energy that would be trapped in the form of a phosphorylated intermediate “X” which would then transfer its “*high energy phosphate*” to ADP by specific enzymes [29]. Initial efforts to identify intermediate “X,” which for a while was thought to be phosphohistidine, failed.

Then, a revolutionary idea was proposed by Mitchell in 1961 [30]. Mitchell's hypothesis was based on the concept known as *vectorial metabolism*, which stated that substrates could be transported across a membrane by an enzyme with a particular orientation against a chemical and electrical gradient. This could only be achieved if it was coupled to another thermodynamically favorable reaction such as ATP hydrolysis. Mitchell's chemioosmotic hypothesis explained mitochondrial ATP synthesis, which in several ways resembles the fuel cell-type, and set the basis for a concept hinging on the coupling of electron transfer to ADP phosphorylation. The supramolecular organization of the enzymes at the inner mitochondrial membrane is an essential component to be considered in this proposal and Mitchell referred to it as anisotropy. In essence, the anisotropic enzymes would act as molecular charge splitters across the inner mitochondrial membrane, in which the redox reactions at the ETS complexes resulted in the separation of protons and hydroxyl anions to each side of membrane, creating the so-called protonmotive force (Δp). As respiration proceeded, the resulting increase in the Δp would drive the separation of protons and hydroxyl anions at the active center of the ATPase, allowing ADP and inorganic phosphate dehydration and ATP formation. In addition, this ingenious proposal offered explanations for many other observations such as (i) the effect of uncouplers, (ii) the regulation of redox state of the ETS components by the magnitude of the electrochemical potential, (iii) the photochemical phosphorylation in chloroplasts, and (iv) the swelling and shrinkage effects on mitochondria associated to the changes in the electrochemical potential. This hypothesis was extensively validated experimentally, being eventually consolidated in 1967 after Mitchell's answers to the criticisms raised by Slater [10]. Although the respiration coupled to ATP synthesis was mechanistically demonstrated, it did not explain how the ATP molecules were produced by the ATPase using the energy accumulated in the form of Δp.

The fundamental basis of the mechanism by which the F_1_F_o_ ATP synthase complex produces ATP at the expenses of the Δp was made possible by the research conducted in the Laboratories of Efraim Racker, John E. Walker, Paul D. Boyer and many others. In fact, a mitochondrial ATPase activity was directly involved in the mechanism by which ADP phosphorylation is coupled to electron transport [31]. This complex was first observed by electron microscopy in the 1960s [32]. Two distinct subcomplexes were seen: one associated to the inner mitochondrial membrane (F_o_) and the other facing towards the matrix (F_1_). Purification of the whole F_1_F_o_ ATP synthase was achieved as well as and the characterization of both F_1_ and F_o_ activities [33]. In 1973, Paul Boyer observed that the exchange of labeled oxygen between inorganic phosphate and water was not blocked by uncouplers of oxidative phosphorylation, but was inhibited by oligomycin (a compound that blocks F_1_F_o_ ATP synthase activity). Interpretation of these results led to the suggestion that ATP might be formed at the catalytic site of this complex. Thus, a significant part of the energy transduced by oxidative phosphorylation is utilized to drive the release of preformed ATP from the enzyme [11]. Later, in 1977, Boyer advanced this concept by proposing an alternating site model for oxidative phosphorylation, in which ATP is formed at one site of the enzyme site, but is transitorily tightly bound. This ATP was not released from enzyme unless ADP and Pi bound a second enzyme site and the ATPase complex became energized [34]. Therefore, net ATP formation by oxidative phosphorylation occurs by a cooperative mechanism involving alternate conformational changes in the *β* subunits of F_1_, promoted by the passage of protons through the F_o_ site of this complex [35].

The passage of protons from the intermembrane space to the mitochondrial matrix mediated by the F_o_ site would drive a rotational movement of the whole F_o_ which, in turn, would transfer the rotation movement to the *γ* subunit and then to the *β* subunits at the F_1_ site. As the interactions between the *γ* subunit and each one of the three *β* subunits are unique, *γ* subunit rotation induces a specific conformation in each of the *β* subunits (open, loose, and tight). When the *β* subunit adopts an open conformation, ATP is released from the enzyme and the active site becomes empty, while the neighboring *β* subunit adopts a *loose* conformation, binding ADP, and inorganic phosphate. Finally, the third *β* subunit is in the closed conformation, expelling water from its active site, allowing the thermodynamically spontaneous ATP synthesis.

### 2.5. Mitochondrial Redox Metabolism

Since a long time, it was known that oxygen played essential biological functions ranging from biomolecule modification to cellular respiration. However, life arose long before oxygen could accumulate in the atmosphere in order to be utilized by cytochrome *c* oxidase. In fact, evidence indicates that organisms in the primitive Earth had simpler metabolic pathways that were not able to fully utilize the energy contained in nutrients. Also, the process of respiration seems to have emerged before the occurrence of significant amounts of oxygen in the atmosphere, as a result of photosynthetic activity. Evidence supporting this interpretation was derived from microorganisms that utilize electron acceptors other than oxygen. Examples are iron, sulfate, vanadium, and even uranium. Along Earth's evolutionary history, organisms that were able to use the sunlight as an energy source to allow water oxidation coupled to molecular oxygen production had a clear advantage. From the energy perspective, oxygen utilization allows a more efficient use of the energy stored in the nutrients through the process of oxidative phosphorylation. Thus, organisms lacking oxygen transport and storage systems, relying simply on its diffusion to the inner parts of its body, exhibited a growth rate that strongly limited by oxygen availability. This idea seems to offer an excellent explanation for the large number of giant fossil records aged approximately 300 million years that lived when atmospheric oxygen levels reached about 35%.

Oxygen is not an inert gas and its toxicity was firstly reported by Paul Bert way back in 1878. He showed that oxygen was toxic to a number of invertebrates as well as fungi, germinating seeds, birds, and even other higher animals. In the central nervous system of mammals, oxygen toxicity was referred to as the “Paul Bert effect.” The mechanisms underlying cellular oxygen toxicity were further studied by Rebecca Gerschman in 1954 who proposed that oxygen potentiated cell death induced by X-ray irradiation [36]. The conclusion was that oxygen and ionizing radiation share mechanisms that possibly involved the formation of “oxidizing free radicals.” A free radical is defined as any atom or molecule that has at least one unpaired electron in an orbital [37]. The term ROS is used to designate not only oxygen-derived free radicals, but also nonradical oxygen species that are capable to generate highly reactive oxygen radicals, such as hydroxyl radical [37]. Because free radicals have unpaired electrons, they tend to achieve stability by donating or removing electrons from adjacent biomolecules such as sugars, lipids, and proteins, resulting in their structural modification. The accumulation of altered or damaged biomolecules by free radicals is associated to a multitude of functional changes in cells, such as apoptosis, mutations, inhibition of enzyme activities, and oxidative stress [37].

Much of the biomedical interest regarding the ROS are due their potential role in the pathogenesis of many diseases and also in aging. In this regard, the seminal work of Harman in 1956, established the well-known “Free-radical theory of aging,” which stated that aging is a result of chronic oxidative modification of biomolecules and structures within the cells that ultimately culminate in death [38]. According to Harman, the cellular free radicals would probably arise by reactions involving molecular oxygen as a result of dehydrogenase activity. Later in 1969, McCord and Fridovich made a central contribution by establishing a link between biology and free-radical chemistry. A copper-containing enzyme, previously identified by Keilin in 1939 as hemocuprein, was found to have a key activity of dismutating superoxide radicals into oxygen and hydrogen peroxide in bovine erythrocytes [39].

Most of the oxygen consumed by the cell is completely reduced to H_2_O by cytochrome *c* oxidase. However, a small portion of this is partially reduced by mitochondria, generating ROS. Complexes I, II, and III of ETS are sources of ROS. In fact, the ETS is not only capable of generating free radicals such as the superoxide radical (O_2_^•−^), but also ROS such as hydrogen peroxide (H_2_O_2_). The first demonstration of mitochondrial ROS formation was made in 1966 by Jensen, who showed that succinate and NADH were able to support hydrogen peroxide formation and this was strongly potentiated by the metabolic poison antimycin A [40]. In the cell, ROS production usually occurs during electron transport along ETS, however, some pathophysiological conditions can increase mitochondrial ROS production because they reduce the activity of ETS or decrease ADP content in mitochondria leading to an increase in the magnitude of the membrane potential, a condition associated with increased ROS “leakage” [41]. Further developments have shown that not only the oxygen pressure, but also the magnitude of the membrane potential strongly affected mitochondrial ROS generation [42]. However, mitochondria are not the only cellular source of ROS. NADPH oxidases, peroxisomes, and endoplasmic reticulum also represent important sites of ROS production.

The imbalance between ROS generation and removal might lead to the so-called oxidative stress. The antioxidant defenses found in biological systems to avoid oxidative stress may be divided into preventive (inhibition of ROS generation), scavengers (suppression of unpaired electrons), and repair (repair of molecules damaged by ROS). The UCPs and hexokinase bound to mitochondria act as preventive antioxidant systems, because both mechanisms aim to reduce membrane potential, promoting mitochondrial depolarization. Cancer cells as well are characterized among other features, by a high expression of hexokinase bound to mitochondria through the VDAC protein. Originally, it was thought that the VDAC bound hexokinase had an exclusive role in the maintenance of the high glycolytic flux typical of cancer cells, but similarly to the mouse brain cells, hexokinase of tumor cells may also maintain the redox balance through an ADP recycling mechanism [43, 44]. The remaining two groups of scavengers are enzymatic (enzymes like superoxide dismutase, catalase and glutathione peroxidase) and nonenzymatic (*α*-tocopherol, thioredoxin). Examples of the repair antioxidant defenses are the poly (ADP ribose) polymerase (PARP) and aldehyde dehydrogenase.

## 3. ROS and Cancer

In the light of Otto Warburg's original observations concerning aerobic glycolysis, it became pertinent to ask whether the mitochondria of tumor cells were functional or not. This question is by no means easily answered and even after 85 years into the post-Warburg era, the issue remains conflicting, or at best riddled with misconceptions. One example of this situation is the oft repeated puzzlement about the cancer cell's selection of the less energy efficient anaerobic glycolysis over the more ATP-rich tricarborxylic acid cycle in conjunction with oxidative phosphorylation. Actually, what cancer cells frequently do is to combine the best of the two pathways in order to sustain the intense proliferation as well as the metastasis that accompanies the transformed state. The glycolytic pathway does not just generate ATP and lactate as the end product. Many of its intermediate metabolites are recognizably anaplerotic in nature as is the case of 3-phosphoglycerate which acts as a precursor to serine, glycine, and cysteine synthesis, all of which are essential for the anabolic condition seen in tumorigenesis. Indeed, it has been recently shown that in human breast cancer, one of the genes that consistently displays a gain in copy number is phosphoglycerate dehydrogenase a result that highlights the association between glucose uptake and aminoacid synthesis [45]. Besides, it must be remembered that glycolysis is a much faster process in terms of ATP synthesis, so that when not hindered by limited amounts of glucose, tumor cells are not badly off. When dealing with the term functionality with regards to mitochondria it must be taken into account that the organelles are a hub to many essential cellular processes. Whilst mitochondria of tumor cells may perform some functions as well as those from normal cells others may be deficient and thus there is a need to specify which of these bear on cancer causally. This question will be addressed below with emphasis on those pathways that are at the same time anomalous and, therefore, amenable to interference by specific inhibitors, especially those connected to the bioenergetics scenario associated to cell transformation.

Functionality of the mitochondria has been addressed in several ways. Many papers dealing with the properties of the organelle, particularly when comparing tumor and normal cells, approach the question in a rather loose and sectored manner. For example, it is common to refer to the physiological status of the mitochondria in terms of its morphology, its respiratory function including ATP synthesis, its role as regulators of intracellular Ca^2+^ homeostasis, or as essential elements in the processes of cell proliferation and apoptosis. Frequently in the literature, the mitochondria are considered functional or not on the basis of analysis that may not be entirely informative of the status of the organelle. For example, in some cases, conclusions about the integrity of mitochondria, or the cell as a whole, are based solely on the ability of certain enzymes to reduce tetrazolium salts and on the assumption that if the enzymes are active, then the organelle and the cells are also viable. Clearly, then, it becomes important to consider what weight should one attribute to these individual parameters and whether they could represent bona fide markers regarding the general health of the mitochondria and sufficient to classify them as dysfunctional. Perhaps a better description for the supposedly aberrant behavior of the organelles within the context of malignant transformation would be “deviant.” The questions that naturally follow this initial discussion are deviant mitochondria are the cause or consequence of cancer? If mitochondria are in fact key elements in cell transformation, which alterations predispose cells to cancer?

### 3.1. Morphology

It is not simple to define what is the normal morphology of mitochondria, mainly because they exhibit considerable plasticity and change their shape radically even when functioning within normal cells. Mitochondria may display transient shape changes as a result of energy demand, that is, oxidative phosphorylation (OXPHOS). A condensed appearance has been associated to mitochondria actively undergoing OXPHOS, whereas the orthodox conformation reflected diminished oxygen consumption. Since in many types of cancer cells the mitochondria have been shown to respire normally, even though with less intensity than glycolysis, it may be difficult to observe morphologies that diverge significantly from the condensed appearance. In other words, the cancer-related morphology of mitochondria may be merely a reflection of the occurrence, or not of the Warburg effect (see below).

Morphology of the organelle refers also to the dynamic processes of fission and fusion with one another and with other organelles that mitochondria undergo in parallel with the cell cycle. Mitochondrial fusion and elongation produces a branched tubular network spreading throughout the cytosol that characterizes what is generally known as mitochondrial networking. Although the mechanism of mitochondria fission, fusion and elongation, is not yet fully understood, some of the key players in this process have been identified and it was the analysis of these components that suggested to a certain degree the distinction between normal and tumor cells. In normal cells, mitochondrial fission occurs in synchrony with cell division. As the cells enter mitosis, mitochondria too begin to fragment, an event which is largely regulated by a GTPase dynamin-related protein 1 (Drp 1), a major component of the fission apparatus. The fission machinery also requires the presence of hFis 1 which is integrated in the outer membrane of the mitochondria. It is thought that interaction between hFis 1 and Drp 1 alters the conformation of the latter leading to the formation of a constricting ring around the mitochondria which ultimately produces fragmentation [46, 47]. In turn, Drp 1 activity itself depends on posttranslational modifications, namely, phosphorylation catalyzed by Cdk 1/cyclin. In addition, the half-life of Drp 1can be modified. It is enhanced by sumoylation by SUMO1 which protects Drp 1 from degradation via proteasome and decreased by deSUMOylation mediated by the protease SenP5. Incidentally, these reactions illustrate quite well how mitochondrial fission responds to elements that normally control the cell cycle and thus becomes synchronous with cytokinesis. Exception should be made to tissues in which cells do not proliferate, such as in muscle. Although the dynamin-related GTPases are the core components of the mitochondrial fission mechanism, recent data have implicated other proteins as upstream regulators of the Drp 1/hFis 1complex and hence of mitochondrial networking [48, 49].

Upon completion of cytokinesis, mitochondria reconnect again through fusion, a complex event that involves the merging of the double lipid membrane of the mitochondria. This is mediated by profusion proteins located on the surfaces of the inner and outer membranes, the complex formed between Mgm1p and the optic atrophy protein OPA1 together with mitofusins (Mfn1 and Mfn2), respectively. The mitofusins also play a role in tethering the mitochondria to other organelles such as the endoplasmic reticulum [50]. This type of interaction does affect Ca^2+^ signaling and exemplifies how mitochondrial networking could have far reaching effects on metabolism as well. By the same token, loss of Mfn2 is known to affect the expression of the subunits that make up the respiratory complexes leading to reduced cellular oxygen consumption. Independently of direct actions of mitofusins on mitochondrial tethering, Mfn2 also regulates the ERK/MAPK signaling pathway a feature that can have a direct bearing on tumorigenesis as it will be discussed ahead [51].

So what is the consensus regarding mitochondria morphology when comparing normal and tumor cells? Within the context of mitochondrial networking, it has been proposed that mitochondria of normal cells spend most of their time in a fragmented mode (postfission state) before they fuse again [52, 53]. Along those lines it has been reported that tumor cells also display a higher frequency of fragmented mitochondria [54], which would indicate the prevalence of fissional events in those organelles. A situation that could be considered analogous would be apoptosis. Reports have shown that when cells are subjected to overexpression of proapototic members of the Bcl-2 family, they exhibit a higher rate of Drp-1-dependent mitochondrial fragmentation and that over expression of fusogenic proteins such as Opa-1 (promoting mitochondrial fusion) protects from apoptosis [55, 56]. Our own results confirm this interpretation indirectly. The mitochondria of H460 cells treated with sodium butyrate, which inhibited cell proliferation, were shown to be more elongated than controls. This was accompanied by a higher expression of Mfn1 suggesting that mitochondrial fusion could be associated to lower rates of cell proliferation [57]. Taken together, the data indicate that except for those cases in which oxidative stress directly induces mitochondrial fission [58], the balance between mitochondrial fusion and fission might depend primarily on the proliferative (or apoptotic) status of the cells. Along those lines, it would be interesting to compare the variations of mitochondrial morphology in different types of synchronized cultures of tumor cells versus that of the normal cell counterparts. *In vivo*, however, there would be experimental complications since it is known that cell doubling time and mitotic indexes are highly heterogeneous even within tumors and also when different types of cancer are compared. In conclusion, so far the morphology of mitochondria cannot be unequivocally associated to cell transformation. The structural mitochondrial alterations observed in many types of tumors cannot be ascribed to any specific neoplasm and this question remains largely unresolved.

### 3.2. Are Mitochondria of Tumor Cells Dysfunctional?

Papers that address the intermediary metabolism of tumor cells typically begin the discussion by quoting the Warburg effect and usually mention that glycolysis replaces mitochondrial oxidative phosphorylation as the principal source of cellular ATP. Frequently, these opening remarks are followed by statements that indicate that the high glycolytic flux adaptation occurs because the mitochondria of tumor cells are dysfunctional or partially disabled. However, the aerobic glycolysis described by Warburg did not imply that the mitochondria were dysfunctional since he himself acknowledged that tumor cells continued to consume oxygen at levels comparable to those of normal cells. In other words, what Warburg really noted was that tumor cells did not exhibit the Pasteur effect, that is, glycolysis in tumor cells persisted even in the presence of oxygen.

The longstanding notion that mitochondria are somehow defective presumably derives from the fact that researchers approach the problem in a nonholistic fashion and frequently assume that if one set of results obtained from tumor cells significantly differs from the normal cell counterparts, other downstream events, even if not investigated, will vary as well. Some metabolic modifications have indeed been detected that when considered individually justified the belief that the mitochondria of cancer cells were somehow impaired. These include the preference for particular respiratory substrates, rates of electron transport, and the activities of enzymes involved in oxidative phosphorylation [59]. Albeit those early reports, data have accumulated to the effect that recently, there has been a shift in opinion. There is now a tendency to accept that mitochondria of cancer cells rarely present defects and seem to retain their capacity to carry out oxidative phosphorylation and consume oxygen with levels comparable to those of normal cells, much as Warburg himself had stated [60]. According to this view, the enhanced lactate production observed in tumor cells does not necessarily imply the cessation of mitochondrial activity, as confirmed by several experiments in which oxygen consumption of cancer cells was evaluated. Such results also indicate some degree of independence between cytoplasmic glycolysis and mitochondrial metabolism acquired by the tumor cells which may explain the absence of the Pasteur effect in cancer cells through the loss of a regulatory interface. In this respect, it was shown that the cytotoxic effect of 2-deoxyglucose (2-DG) on A549 lung cancer cells depended on inactivation of mitochondria in p53^−/−^cells. The observation that the Warburg effect was only evidenced in cells in which mitochondria were impaired supported the idea of a functional link between glycolysis and the oxidative reactions of the organelle [61]. Furthermore, the often quoted idea that in cancer cells the mitochondria stop respiring in order to save carbon skeletons for the biosynthesis of other biomolecules required for rapid growth is not compatible with the observation that tumors actually excrete high amounts of lactate [62]. That is not to say that the macromolecules and lipids found in the intramitochondrial milieu do not display alterations that while observable may not significantly compromise mitochondrial respiratory function. The enhanced production of reactive species of oxygen (ROS) and reactive nitrogen species (RNS) associated to cancer cells (discussed in Section 3.3) can definitely cause damage to proteins, DNA, RNA, and membranes. However, the mutations produced in mtDNA are not necessarily synonymous with tumorigenesis. It is known that germline mutations of mitochondrial DNA can cause diseases that affect children and adults ranging from mitochondrial myopathies to retinitis pigmentosa and possibly even to autism, but not cancer. In an analogous manner, sporadic mutations that may result from oxidative damage due to elevated ROS in the mitochondria are suggested by many to be the culprits of tumorigenesis. This has been difficult to demonstrate, however, mainly because there is no strong evidence showing that the mtDNA mutations are driver mutations. Also, the majority of somatic mutations found in mitochondrial DNA are not harmful to the cells and populational surveys showed that the so called specific cancer mutations (varying from 30–100%) are frequently found in mitochondrial DNA of individuals with no history of cancer. Hence, it is thought that ROS-induced mtDNA mutations may actually occur as a consequence of metabolic reconfiguration of cancer cells. Nevertheless, papers abound that correlate the mitochondrial mutations to several types of cancer [63].

How to resolve this quandary? There is a very tight connection between the nuclear and mitochondrial genomes coordinating the expression of exons encodings different subunits of proteins making up the electron transport chain. Thus, it would be desirable to separate the individual contributions of nuclear and mitochondrial DNA mutations to the cancer phenotype. After the advent of the technique of cytoplasmic hybrids (cybrids), or transmitochondrial cybrids, it became possible to repopulate cells from which mitochondria had been depleted, with exogenous mitochondria present in enucleated cells. In this manner, the information obtained from the fused cells, for example, cancer cells, should highlight which events could be assigned to the mitochondria alone [64]. Results obtained with this experimental approach have demonstrated that many of the respiratory alterations ascribed to mitochondria of cancer cells (and other pathologies) could be reproduced in the “transplanted” cells. For example, Imanishi and collaborators [65] were able to show that mitochondrial respiration defects observed in human breast cancer cells caused by mtDNA mutations were responsible for the expression of high metastatic potential in recipient cells. Interestingly, these experiments with cybrids demonstrated that the mutated mtDNA affected metastasis, but not cell transformation. Results that confirmed that the increase in metastatic potential was acquired were also obtained in a mouse model in which the transferred mtDNA had a mutation in the NADH dehydrogenase subunit 6, a deficiency that augmented the production of ROS. In these experiments, the metastatic potential of the cybrids was enhanced and use of a scavenger such as N-Acetyl cysteine was able to counteract it [66]. Correlations exist showing that ROS and RNS are able to inhibit the process of anoikis, that is, the occurrence of apoptosis as a result of loss of cell adhesion. In normal cells, anoikis prevents detached cells from colonizing different tissues. According to this, the oxidative stress generated by ROS and RNS overproduction in tumor cells would favor metastasis by means of activation of prosurvival signaling pathways that in turn would inhibit anoikis and in this manner boost the progression of cancer [67]. It would be very interesting if it could be demonstrated that alterations in the mtDNA alone and by extension the higher production of ROS could be responsible for metastasis. Other parameters such as diminished cellular oxygen consumption and ATP synthesis observed in human breast cancer cells could also be successfully passed onto the cybrids and thus were firmly interpreted as derived from mitochondria [68]. Diminished respiratory rates, except when connected to damage or reversal of the electron transport chain, however, would generate less ROS a result which would go against the grain according to the canonic view of ROS-induced tumorigenesis.

Although with the aid of the cybrid technology, the role of mitochondria in tumorigenesis could be better appreciated, caution should still be exercised to avoid misinterpreting, or overstating certain selected parameters. Some of those have limited information. For instance, when studying mitochondrial respiratory control, values for the P/O ratio, a very popular analysis, do not really define whether mitochondria are functional or not since on its own they reflect proton leak as a result in fluctuations of states 3 and 4 of respiration. Others, as in the case of experiments using isolated mitochondria, may result from manifestations that occur independently of the regulatory grid that normally control the organelles. Thus, experiments generating data based on morphology (see Section 3.1) as well as evaluation of reactions to specific stressors have to be interpreted with reserve because they abrogate the innate responses connected to HIF-1*α* and hypoxia. In addition, when using intact cells to investigate mitochondrial function, researchers have to make use of detergents such as digitonin in order to allow access of cell-impermeant substrates to the organelle. This experimental resource may produce artifacts due to damage to the mitochondria outer membrane resulting in the release of components such as cytochrome *c* that in turn may trigger cellular responses independently of the bioenergetics analysis being carried out. In a recent review, those questions have been clearly and carefully dissected pointing out the pros and cons of each approach [69].

The importance of mitochondria as fully functional organelles in cancer cells has been strengthened by considering the recently proposed hypothesis that metabolically they actually conform to the orthodox model of ATP production via the regular set of mitochondrial oxidative reactions, like the TCA cycle, oxidative phosphorylation, and the anaplerotic glutamine utilization pathway. According to this empirically based hypothesis, cancer cells are not considered as rogue cells that become immortalized and manage to live independently of other tissues. Reports have described a lactate shuttle that is formed between stromal cells and the cancer cells, in which the former predominantly glycolytic, feed the latter, the oxidative tumor cells that utilize mitochondria to their full capacity. This hypothesis maintains that aerobic glycolysis, the hallmark of many tumors, is actually carried out by the cancer associated fibroblasts rather than the cancer cells themselves [70, 71]. With the metabolic symbiosis thus established, the stromal fibroblasts would undergo autophagy and mitophagy and as a result secrete and supply lactate to the cancer cells. In turn the fibroblasts would profit from the available mitochondria in the latter. This phenomenon was termed the “reverse Warburg effect” and the autophagy/mitophagy occurring in the stromal fibroblasts would be induced by oxidative stress triggered by the cancer cells. The proponents of this model went as far as mentioning that, in some cases, the Warburg effect might in fact represent an *in vitro* artifact. Although a similar metabolic symbiosis seems to occur in normal cells, it remains to be demonstrated whether the reverse Warburg effect could be extrapolated to other types of cancer [72]. At any rate lactate fueled respiration, a feature of metabolic symbiosis, has been demonstrated in tumor cells in mice, thus strengthening the idea of the interdependence that exists between normal and tumor cells [73].

In conclusion, it can be safely stated that the mitochondria of tumor cells are functional and that they may have a significant role in the maintenance of proliferation and metastasis.

### 3.3. ROS and RNS Production in Tumor Cells

This section tries to appraise the role of ROS and RNS in cancer formation. At the same time, it introduces the question whether the oxidative stress signature of cancer cells can indeed be a prime target for therapy. ROS and RNS can be regarded as decidedly toxic to the cells by considering many of their direct or indirect effects on biomolecule targets. However, toxicity, or for that matter the adjuvant role in carcinogenesis that ROS and RNS have appeared to depend ultimately on their final concentrations at a given instant and on the duration of the stimulus. In consequence, ROS and RNS levels depend on the regulation of the pathways that generate them as well as those that degrade them (scavengers). When ROS and RNS concentrations are within a certain range of the concentration gradient, “physiological” steady-state levels of cellular ROS and RNS perform the housekeeping coordination of metabolic and genetic processes. As such H_2_O_2_, for example, stimulates cell proliferation by acting as modulators of various transcription factors that in turn influence several important cellular processes. Among the transcription factors, NF-*κ*B, Nrf2, p53, HIF-1*α*, and STAT3 could be mentioned. The mechanism whereby ROS modulate transcription factors involves the reversible oxidation of cysteine residues of proteins belonging to signaling pathways such as protein tyrosine kinases and phosphatases, lipid phosphatases, proteases, and signaling effectors. Exposure of proteins to the oxidative environment generates dityrosine residues that can be considered as oxidation markers. Apart from its effect on proliferation, H_2_O_2_ also mediates cell differentiation and migration. As opposed to the physiological role of ROS in normal cells, the oxidative stress is characterized by situations in which the levels of molecular oxygen or its ROS derivatives increase above the threshold of normality and produce widespread irreversible oxidation of aminoacids, polydesaturation of fatty acids, and mutations on DNA and RNA. Mutations in nucleic acids occur by formation of C5-OH and C6-OH adducts of thymine and cytosine or similarly with purines, or by reaction with the sugar moiety of the polymer leading in some cases to single or double-strand breaks, intrastrand cross-links, and protein-DNA cross-links [74–76]. This destabilizing effect of ROS on DNA can promote genomic instability which as a consequence may predispose the cells to malignant transformation. In this context, cancer cells that are able to survive in a supposedly hostile microenvironment of high ROS and RNS could be regarded as a subpopulation that were selected in terms of their peculiar metabolic adaptations. This view, however, is not without controversy. Before discussing the possible mechanisms of adaptation, it is relevant to inquire whether there is a consensus concerning the ROS associated etiology of cancer.

What then, if there is a pattern, is the ROS/RNS phenotype of cancer cells? Are ROS and RNS able to act as stimulants of cell transformation cells by subverting the normal control network through a sustained oxidative environment, or do they act directly producing harmful modifications on lipids, proteins and RNA/DNA? Or both? The earlier work of Szatrowski and Nathan [77] showed that relatively large amounts of hydrogen peroxide, comparable only to polymorphonuclear leukocytes and monocytes, were produced by a number of tumors. Since then, the list has grown and many reports indicate that cancer cells produce massive amounts of ROS to levels that exceed the capacity of the antioxidant enzymes that are normally in charge of ROS detoxification. Adding to this, there is evidence showing that excessive ROS production causes the progressive inactivation of the antioxidant enzymatic systems, a condition that favors the maintenance of high concentration of ROS and the induction of a chronic oxidative state. Results published by several groups indicate that it is this situation that sets the scene for transformation. According to this hypothesis, the maintenance of parameters such as cell proliferation within the boundaries of normalcy would depend primarily on the redox balance established between ROS production and the activity of antioxidant enzymes such as superoxide dismutase, glutathione peroxidase, glutathione reductase, catalase, and thioredoxin as well as the nonenzymatic antioxidants scavengers glutathione and vitamins C and D.

In contrast, increased levels of ROS, that may result from a failure of the antioxidant system rather than from overproduction, have been associated to chronic degenerative conditions such as cancer, aging, diabetes, and cardiovascular diseases [78, 79]. The site of the oxidative stress has been narrowed down to mitochondria and was covered extensively in a recent review [80] that highlights the prooxidant role of the altered organelles as protagonists of tumorigenesis. Corroborating this view, the high levels of ROS associated to the pathophysiology of a wide variety of cancers have prompted clinicians to examine the amount of dityrosine residues in proteins exposed to an oxidative environment. Proteins that are highly susceptible to oxidative attack have in fact been termed as advanced oxidation protein products (AOPP) and currently serve as markers for monitoring cancer patients [81]. Numerous other reports have generalized the notion that cancer cells are more challenged by oxidative stress than normal cells [82]. Among the types of cancer that have been linked to increased oxidative stress are bladder, brain, breast, cervical, gastric, liver, lung, melanoma, multiple myeloma, leukemia, lymphoma, ovarian, pancreatic, and prostrate [83]. In a number of reports, it has been found that genes involved in oncogenic pathways and those linked to tumor suppression are frequently mutated in transformed cells all of them sharing the common feature of increased amounts of ROS [84–88]. Finally, the hypothesis that ROS and RNS are early effectors of tumorigenesis is in agreement with data revolving around the inflammatory reactions. A hegemonic view on the etiology of cancer states that when an inflammatory condition lasts long enough it behaves as a prodrome to cancer. The normal course of an inflammatory reaction produced by a number of different agents including bacteria, carcinogens, and radiation progresses to a stage in which mast cells and leukocytes are mobilized to the sites of lesion. These cells produce what could be described as respiratory bursts that in turn contribute towards the increase in local production of ROS. The respiratory bursts are actually amplified several fold by other inflammatory cells attracted to the site of inflammation by chemokines and cytokines released by the former cells, so that the oxidative stress actually propagates to neighboring cells. In this context, it is known that the extent of tumor associated macrophage infiltrates correlates well with the prognosis of certain types of cancer. If inflammation is not reversed, it could then create a vicious circle whose outcome is chronic ROS-induced oxidative stress and ultimately, cell transformation. The occurrence of inflammatory reactions with the participation of cell infiltrates in premalignant senescent hepatocytes has also been demonstrated in inflammation-based mouse models of hepatocellular carcinoma [89].

One question could be raised concerning the increase in ROS as an oncogenic factor. Why, in view of the known roles of high ROS in inducing senescence and apoptosis, cancer cells do not undergo apoptosis? Presumably in these transformed cells the pathways connected to senescence and apoptosis are somehow blocked. One proposed mechanism for this inactivation involves p38 MAPK pathway. In normal cells, it is known that elevated ROS induces apoptosis via the p38*α* MAPK. In contrast, human cell lines in which p38*α* is inactivated are refractory to ROS-induced apoptosis which suggests that deficiencies in this pathway, as well as those which involve p53 (often mutated in most cancers) allow cancer cells to remain viable in the presence of high ROS [90]. The question whether cancer cells survive in a hostile ROS environment as a result of enhanced activity of antioxidant enzymes and compounds must also be considered.

Many papers attest to the fact that tumor cells are usually well adapted to high levels of ROS and that their viability is only possible due to enhancement of antioxidant activity [60]. However, it should be borne in mind that tumor cells, which occur in a tumor in different stages of transformation, should exhibit heterogeneous metabolic profiles. This would be due to oncogenic gain-of-function and/or loss of tumor suppressors. These alterations may generate a mosaic of metabolic patterns resulting from differentially expressed enzymes which in the same tumor would reveal increased ROS levels and downregulated antioxidant systems. Such a scenario is compatible with the model of waves of gene expression proposed by Smolková and collaborators [5].

As alluded some findings cannot be generalized to all types of tumors and to all situations. Some points that require further discussion are listed below.

(a) *Source of ROS*: scientists do not agree as to the source of ROS. Many reports state flatly that mitochondria are the main producers of ROS and highlight that organelle as the site where the tumorigenic process begins [91]. Others call attention to the fact that there are alternative and perhaps more important intra- and extracellular ROS generating reactions that include the endoplasmic reticulum, peroxisomes, the cytosol, plasma membrane, and the extracellular space [92]. Among the most important nonmitochondrial sources of ROS is the NADPH oxidase family of enzymes. These are bound to the plasma membranes and to the membranes of phagosomes. Of the seven oxidases that comprise the NADPH family, Nox1, Nox2, and Nox4 are expressed by several types of cancer cells, including colon, prostate, gliomas, melanomas, pancreatic adenocarcinomas, renal carcinomas, and ovarian. Other incidental nonmitochondrial sources of ROS include those that are generated by external factors such as carcinogens and radiation that are known effectors of inflammatory reactions that usually precede cell transformation (see below).

In the context of cancer, the NADPH oxidases should be highlighted not only because of their quantitative contributions to ROS formation, but also as promoters of tumor-induced angiogenesis, an important process adjuvant to tumor growth and metastasis [93]. Supporting the role of NADPH derived superoxides as promoters of tumor induced angiogenesis, experiments have shown that antioxidants such as vitamins C and E reduced the expression of vascular endothelial growth factor (VEGF) and vascular endothelial growth factor receptor 2 (VEGFR-2) in mice [94]. Likewise, *in vivo* experiments demonstrated that the scavenger NAC was able to inhibit sarcoma-induced angiogenesis, thus strengthening the ROS-induced tumorigenesis model [95].

(b) *Are ROS tumorigenic? *There is a growing consensus as to the role of ROS in tumorigenesis. In many works, ROS are clearly singled out as the main tumorigenic factors [96, 97]. Some reports, however, indicate that ROS are harmful and tumor cells are only able to survive thanks to efficient or exacerbated antioxidant defense systems [98, 99]. Put another way, mitochondria of tumor cells, which as mentioned above are believed to be fully functional and which according to many are the main producers of ROS, play the simultaneous role of providing energy to the cells, and also of introduce the tenuously controlled oxidative stress, the sword of Damocles as it were [70]. Interestingly, ROS production seems to be much higher in mitochondria in which damage to the respiratory chain occurs, or mitochondria that are actively undergoing reverse electron transport, than those that are normally producing ATP [100]. This situation illustrates then how the processes of autophagy and mitophagy are beneficial to the cells as removers of potential sources of cell transforming ROS [53]. Adding support to the toxic effect of ROS to cancer cells, it is worth mentioning that both, radiotherapy and chemotherapy, normally employed to eradicate cancer cells do so by producing ROS-mediated oxidative stress [97]. Although it is known that ROS have multiple downstream effects, what is emerging as an explanation to conciliate all the conflicting data is that the overall outcome of the ROS-triggered activations/inhibitions depends primarily on their final concentration. In order to even out the data in the literature that show a wide variation in ROS concentrations produced by different types of tumor cells, it would be important to show whether the observed oscillations are actually due to the activities of antioxidant enzymes or the presence of higher levels of free radical scavengers in these cells. This seems to be the case of cancer stem cells that appear to have lower level of ROS than normal cells [101]. Likewise, hematopoietic systems display low levels of ROS although the progenitor cells myeloid produce high levels of ROS. If the hypothesis of antioxidants as the regulators of ROS and hence as pace makers of tumorigenesis proves to be right, the management of cancer should afford many strategies of interference based not only on the use of antioxidants, but on inhibitors acting on ROS downstream signaling pathways. Distinction should be made among the ROS, however. Nitric oxide (NO), for example, has been shown to have antiproliferative effects in both, normal and tumor cells [102, 103], and it has been demonstrated that superexpression of nitric oxide synthase (iNOS) caused inhibition of proliferation of pancreatic tumor cells. When considering the effects of ROS on transformed cells, another issue has to be taken into account which involves the plasticity of tumor cells regarding exposure to ROS. Results of experiments in which tumor cells in culture were incubated in the presence of increasing amounts of ROS showed that they responded by displaying an enhanced tolerance to the oxidative stress. Results obtained by Onul et al. [104] evidenced further that A549 lung cancer cells adapted to long-term high levels of hydrogen peroxide grew better in culture than the parental cell line and were more resistant to the chemotherapeutic agent Doxorubicin. Interestingly, those adapted cells definitely favored a more anaerobic metabolic profile suggesting that the survival strategy adopted might be independent of mitochondria. The same standing applies for high concentrations of nitric oxide (NO). In breast tumor cell lines, high concentrations of NO were shown to induce a phenotypic change [105]. Thus, it may well be that it is the metabolic adaptation of tumour-initiating cells that could dictate the development of cancer rather than HIF activation or other signaling pathways that have been known to affect solid tumor growth. Whether the cells adapted to high ROS would be resistant to antioxidant therapy remains to be investigated. In conclusion, the individual contributions of different cell compartments to the final ROS concentration within the cells may predispose them to transformation, mainly in those cases when the antioxidant systems are not effective in counteracting the oxidative stress. Excess of ROS is also harmful to cancer cells.

### 3.4. Different Tumors, Different Biochemistries

Attempts to build a grand unifying model of metabolic reprograming in cancer cells that would make biochemical sense are perhaps premature. As it became clear from the preceding discussion, the available data do not always fit into coherent mechanisms and so far ideas do not converge to a single stratagem to combat cancer cells. Apart from the controversies, experimental difficulties, and conflicting interpretations, there are inherent differences that have to be considered before one draws a standard biochemical profile analysis of different tumors. Firstly, normal tissues have individual metabolic rates that would certainly influence the type of metabolism occurring in the cognate transformed cells. Slow-twitch and fast-twitch muscle tissue, for example, obtain ATP preferentially from different pathways. The same applies for several other tissues in which the metabolic diversity reflects the presence of specific isoforms of enzymes, as occurs in liver and brain tissue. It is plausible then to imagine situations in which as a function of distinct metabolic rates, variable levels of ROS would be produced that could cause different types of local lesions. Secondly, individuals are themselves different when considering their biochemical buildup. The emerging field of pharmacogenomics recognizes these differences and the trend is now, taking advantage on available high throughput technology, to carry out individual genome and transcriptome analysis for patients undergoing long term chemotherapy. However, biochemical diversity seems to transcend genes. Population studies have suggested that the majority of cancers is in fact of the sporadic type and hence would reflect the life style of individuals more than their genetic background. So pharmacogenomics must be complemented by “pharmacometabolomics” which could be adapted to the individual needs according to the redox profile of the tumors under treatment. ROS-based treatment would thus select combinations of prooxidant and antioxidants agents that would best counteract the anomalous oxidative stress generated, preferably at the transformative stage. In other words, the prooxidant, and antioxidant medication would be tissue tailored. That the antioxidant, system renders itself as a target for chemotherapy was eloquently shown by Raj and collaborators [99] using a murine model. In this work, cancer cells from different types of tumors, but not normal cells, were effectively killed by piperlongumine, a small molecule derived from the plant *Piper longum*. In addition, the authors were able to demonstrate that the cytotoxic effect of piperlongumine was achieved through interference with the antioxidant systems of the tumor cells. Such an approach has the added advantage that inhibitors targeting antioxidant systems could also be prescribed on a preventive setting.
